# Optimization of Mechanical Structure for Pomegranate Peeling and Low‐Damage Seed Extraction Technology Based on Mechanical Property Analysis: A Comprehensive Review

**DOI:** 10.1155/ijfo/5503722

**Published:** 2026-05-16

**Authors:** Enlong Zhu, Jinkun Ran, Li Li, Junwei Zhu

**Affiliations:** ^1^ Department of Mechanical Engineering, Tianjin University of Science and Technology, Tianjin, China, tust.edu.cn; ^2^ Tianjin Key Laboratory of Integrated Design and Online Monitoring of Light Industry and Food Engineering Machinery and Equipment, Tianjin, China; ^3^ Tianjin Ruijing Tianyuan Farm, Tianjin, China; ^4^ YanShan University, Qinhuangdao, Hebei, China, ysu.edu.cn

**Keywords:** peeling mechanical structure, peeling scheme, structure optimization, texture characteristics, Xinjiang pomegranate

## Abstract

With a rich history of cultivation, pomegranates are an essential cash crop planted extensively, offering considerable economic gains in the fruit industry. Within the pomegranate processing industry, pomegranate processing equipment enhances seed extraction efficiency and liberates labor. This paper reviews the current state of development in pomegranate processing technology and equipment both domestically and internationally, covering various mechanical structures, the current application status of equipment, and the principles of mechanical peeling mechanisms. It analyzes the limitations present in the existing pomegranate processing equipment. This study explores key technical pathways for automating pomegranate peeling and seed extraction. It encompasses conducting mechanical property tests to determine physical parameters and mechanical characteristics, designing an adjustable mechanism, and establishing preprocessing steps for removing calyx and leaves. Consequently, a multistage pomegranate seed extraction technical solution incorporating preprocessing procedures is proposed. This method involves removing the calyx leaves from pomegranates to reduce the difficulty of opening them, thereby increasing seed extraction rates and minimizing seed damage. The calyx removal success rate exceeds 98%, with pomegranate damage rates ≤ 5%. This study provides guidance for the upgrading of the pomegranate processing equipment industry.

## 1. Introduction

Pomegranate (*Punica granatum*) is the fruit of pomegranate, which is a kind of excellent fruit with ecological, economic, and social benefits, ornamental value, and healthcare function [1]. It originated in Central Asia and spread eastward to India and China, and westward to countries around the Mediterranean Sea and other suitable habitats in the world [2]. At present, studies on pomegranate have found that pomegranate peel contains bioactive substances such as phenols and flavonoids [3]. Its extract polyphenols, ellagic acid, and punicalagin have high antioxidant and antibacterial effects and can effectively delay the growth of micro‐organisms such as molds and bacteria in food [4]. Tannin is the main material for tanning leather. Pomegranates contain 10%–25% tannins, with pomegranate leaves containing 11% and pomegranate peel containing 26%. The fruit pulp left over each year from Morocco’s juice industry serves as a valuable resource for extracting tannins [5, 6]. Pomegranate fruit can be made into fruit juice, jelly, jam, or fruit wine. Its market and industrialization prospects are very broad [7]. The medical value and economic value of pomegranate have promoted the vigorous development of pomegranate deep‐processing industry.

When pomegranate blossoms, the outer side of its petals is wrapped by calyx. The calyx is relatively hard and thick, with a fleshy tubular top sharp, connected to the ovary, and the arrangement is tile‐shaped. The sepals are part of the calyx, usually triangular, hard, and thick, with a length of 2–3 cm and a thickness of about 2–3 mm. Their main function is to protect the petals and buds [8]. The thickness of the peel is about 1.5–4 mm, and the peel is wrapped with pomegranate seeds, usually red or yellow, and the texture is tough, which can effectively protect the pomegranate seeds and aril inside the fruit. The storability and shipping durability of pomegranates are largely governed by the physical properties of the peel, specifically its thickness and toughness. The placenta is the part connecting the aril and the pulp. The structure of the placenta is usually sponge‐like and has multiple protrusions. The protrusions connect the pomegranate seeds, which provide nutrition and support for the seeds. There are persistent calyxes on the top of pomegranate fruit. The peel is coated with juicy seed coat. The seeds are separated by white astringent thin septum. The color of pomegranate peel changes from yellow, green to deep red according to different varieties [9].

Referring to the annual data of the Food and Agriculture Organization of the United Nations (FAO), the United States Department of Agriculture (USDA), and the national agricultural sector in recent years, from 2017 to 2021, the average annual compound growth rate was 2.1% [10, 11]. From 2021 to 2024, the global production of pomegranate increased year by year. In 2024, the global production of pomegranate was about 6 million tons, and the global market size of pomegranate was $299.56 million [10]. The main producing countries accounting for 75% of the total global production were India, Iran, Turkey, China, the United States, Israel, and so on [12]. China’s pomegranate production has remained stable at over 1 million metric tons from 2021 to 2023. China has formed eight major pomegranate‐producing areas: Lintong in Shaanxi, Zaozhuang in Shandong, Xingyang and Kaifeng in Henan, Huaiyuan in Anhui, Mengzi in Yunnan, Huili in Sichuan, and Yecheng in Xinjiang. The existing pomegranate planting area in Xinjiang is about 2 × 10^4^ hm^2^. It is mainly concentrated in Pishan County in Hotan Prefecture, and Yecheng County and Shufu County in Kashgar Prefecture. According to the data of the National Bureau of Statistics in 2022, the cultivated area of pomegranate in Shaanxi Province was 4.45 × 10^3^ hm^2^ and the yield was 7.17 × 10^4^ tons; the annual output of pomegranate in Xinjiang reached 3.46 × 10^5^ tons, accounting for 68% of the total output of the country, of which Hotan area accounted for more than 70% (2.42 × 10^5^ tons). The demand for pomegranate processing equipment is imminent, and the research in the field of pomegranate processing is particularly important [13].

Pomegranates feature thick outer skins rich in tannic acid, while their delicate seeds are prone to damage [14]. Manual peeling and seed extraction is an extremely tedious and time‐consuming process, requiring a large number of skilled workers. This results in labor costs accounting for a disproportionately high share of total production expenses, becoming a critical bottleneck constraining the entire industry’s development. Manual processing struggles to quickly and thoroughly remove the outer skin and separate the seeds, facing a clear efficiency ceiling. Manual handling inevitably causes pulp damage and juice loss, while inconsistent hygiene standards lead to significant quality issues, including heightened food safety risks [15]. Automated pomegranate processing equipment significantly reduces costs compared to manual labor and operates at a notably higher efficiency than manual processing (one machine can process one ton of pomegranates per hour). The pomegranate processing industry is undergoing a transition toward automation [16].

**FIGURE 1:**
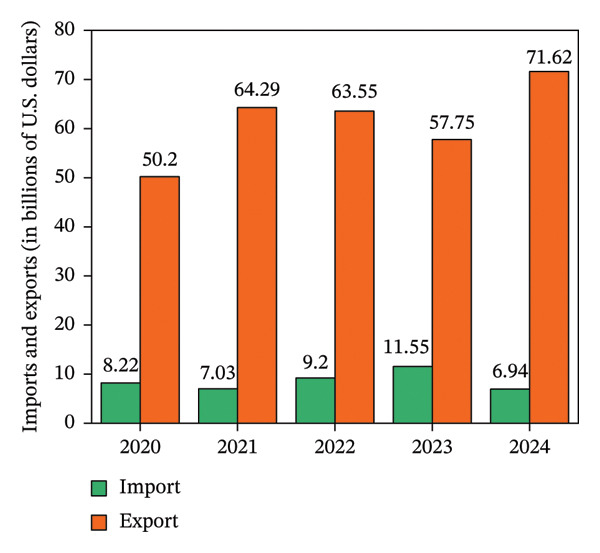
The import and export amount of China’s agricultural machinery from 2020 to 2024 (the unit is billions of dollars). Reproduced with permission from Ref. [10].

From 2020 to 2024 (Figure 1), China’s agricultural machinery imports and exports increased annually, with imports reaching $71.62 billion in 2024. The agricultural machinery market holds vast potential for growth. With expanding demand for pomegranates and the upgrading of pomegranate processing products, research and development of pomegranate processing equipment is accelerating, including advancements in visual positioning and flexible gripping mechanisms. Mechanized peeling technology has gradually become a research hotspot. The existing mechanical peeling methods include high‐pressure water jet cutting, mechanical peeling, and impact‐based cracking. Compared to manual seed extraction, these techniques not only enhance peeling efficiency but also reduce pomegranate seed breakage rates. Therefore, the development of pomegranate peeling machinery not only is driven by the need to enhance production efficiency but also represents a crucial measure for promoting the sustainable development of the pomegranate deep‐processing industry.

This study systematically evaluated the technological innovation of pomegranate processing through three progressive objectives: (1) to evaluate the effectiveness and influencing factors of the existing peeling and threshing system; (2) by studying pomegranate‐related properties, elucidate the understanding of pomegranate structure, combined with the existing peeling scheme to analyze the mechanism of pomegranate deconstruction threshing; and (3) based on the current progress, a targeted optimization scheme is proposed for the industrial application of pomegranate.

## 2. Peeling Machine Design Proposal

The peeling process of fruits and vegetables is an important part of food processing, and its mechanical structure design directly affects the peeling efficiency and the quality of fruits and vegetables. Modern peeling machines use a variety of techniques such as blade cutting, friction peeling, extrusion deformation peeling, and water jet peeling. These mechanical structures not only achieve efficient peeling functions but also reduce damage to fruits and avoid pollution and nutrient loss [17]. Therefore, an in‐depth study of the structural design and working principle of fruit and vegetable peeling machinery will provide an important theoretical basis and technical support for improving food processing efficiency and ensuring food safety.

### 2.1. Cutting Blade Proposal

The cutting method of the cutting tool is to use the sharp cutting tool to cut the fruit directly, which is simple and fast. The cutting tool is suitable for thin‐skinned fruits and vegetables and end‐connected fruits [18]. Rotary blade cutting peeling uses a blade to fit the rotating fruit in the clamping to remove the outer skin of the material. The cutting depth can be controlled by adjusting the rotation speed of the blade, which is suitable for large‐scale industrial production [19]. If it cannot adapt to the same type of fruit of different sizes, it will cause obvious mechanical damage to the fruit, affecting the quality of the product and the length of preservation.

When the tool peeling scheme is applied to pomegranate, it is necessary to consider the characteristics of irregular fruit shape, uneven skin thickness, and grain vulnerability. Although the existing rotary cutting method has high efficiency, it may cause excessive cutting or damage to the grain due to the size difference and surface bumps of pomegranate [20]. The flexible cutting mechanism that can respond to the change of fruit shape in real time can be developed by referring to the profiling peeling and constant force adaptive design, so as to reduce the mechanical damage and maintain the integrity of pomegranate seeds while ensuring the peeling efficiency, so as to meet the needs of high‐quality processing.

**FIGURE 2:**
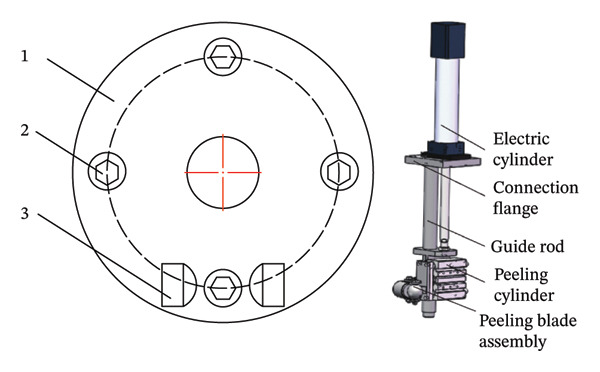
Peeler structure reproduced with permission from Ref. [21]. 1: electric cylinder, 2: turntable fixture, and 3: U peeling knife of type.

According to the adaptability of peeling machine to different sizes of fruits and vegetables, Guohong et al. [21] designed a flexible adaptive profiling peeling mechanism. The verification test of sweet potato peeling performance showed that under the optimized working parameters, the peeling effect of the sweet potato peeling machine was better. The average peeling time of each sweet potato was 10 s, and the working efficiency was 360/h, which ensured the consistency of the peeling thickness of sweet potato and the stability of the peeling operation, and met the industrial processing requirements of sweet potato peeling [21] (see Figure 2).

The factors affecting the peeling machine of the tool include the material of the tool, the degree of sharpness, and the speed of the motor [22]. The degree of sharpness has a significant effect on the peeling efficiency. Excessive cutting force will lead to serious damage to fruits. Too fast cutting speed may lead to incomplete peeling, and too slow will reduce peeling efficiency. For objects with irregular surfaces, it is necessary to design profiling tools [23]. The cutting angle and shape of the tool affect the smoothness of the cutting and the removal effect of the cortex [24]. A reasonable tool angle can reduce the surface damage of the object. Too fast feed speed will lead to uneven cutting, and too slow feed speed will reduce production efficiency.

### 2.2. Friction Peeling Proposal

Friction peeling removes the skin by generating friction on the surface of fruits and vegetables [25]. This technology is especially suitable for thin‐skinned fruits and vegetables such as peaches and tomatoes. Friction peeling technology development so far, mainly includes two kinds of designs: brush friction peeling and vibration friction peeling. The brush friction peeling is mainly equipped with a rubbing roller and a brush mechanism [26]. The rubbing roller adopts a floating design to adapt to different sizes of fruits and vegetables. Its surface is made of flexible and high‐wear‐resistant rubber to reduce damage to the fruit. Vibration friction peeling uses vibration equipment to allow materials to continuously collide and friction in the workspace, thereby removing the outer skin layer [27].

**FIGURE 3:**
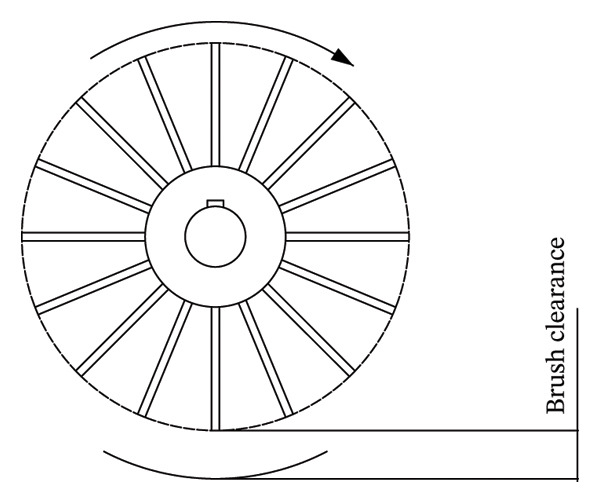
Schematic diagram of friction wheel mechanism. Reproduced with permission from Ref. [28].

Xinping et al. [28] designed a modular flexible garlic peeling device. Garlic peel separation is achieved through a floating friction unit, while material conveyance is accomplished using a vibration unit. Flexible peeling is realized through the combined action of the floating friction unit, vibration unit, combing brush unit, and airflow blowing unit. The clove peeling rate reached 93.68%, with a damage rate of 4.40% (see Figure 3).

The material, hardness, and roughness of the friction material will significantly affect the peeling effect, and the rougher or leathery objects are easier to peel [29]. For example, the friction material with uneven and rough surface has better peeling effect when dealing with soft‐skinned fruits, and excessive pressure will lead to the removal of cortex and pulp. Fruits with harder peels require higher hardness materials or greater pressure to increase friction, and too small peels are not completely removed. The friction force directly affects the removal efficiency of the cortex, and the rotation speed affects the uniformity and efficiency of the peeling.

### 2.3. Crushing and Extrusion Peeling

The mechanical extrusion deformation peeling device primarily separates the skin from the flesh of fruits or vegetables through mechanical extrusion, particularly for those with thicker, tougher, or fibrous skins [30]. This type of device efficiently removes the skin while preserving the integrity of the flesh, avoiding the fruit flesh loss or damage that may occur with traditional peeling methods [31]. The basic principle of the extrusion deformation peeling machine is to apply external force via mechanical devices, causing the object to deform or rupture, thereby achieving the separation of peel from pulp [32]. The principle of extrusion deformation peeling mainly relies on physical and mechanical actions, such as pressure, shear, and friction, to detach the skin from the flesh.

**FIGURE 4:**
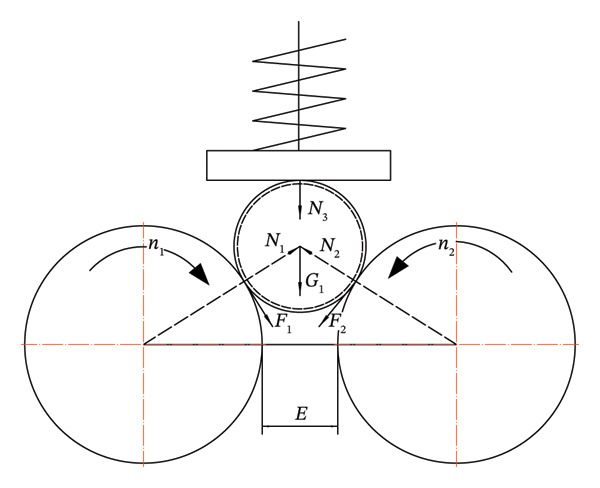
Macadamia nut stress analysis diagram. Reproduced with permission from Ref. [33].

Chen et al. [33] optimized the double‐roller peeling equipment for Satsuma mandarins, primarily employing a “compression + friction” separation principle. The optimal parameters were determined as follows: hot steam heating for 3 min, roller rotation speed of 60 r/min, and plate translation speed of 12.98 mm/s. Under these conditions, the peeling rate reached 100%, while the damage rate was 1.96% (see Figure 4).

### 2.4. Water Jet Peeling

Water jet peeling is a sophisticated method of surface treatment that uses high‐pressure water jets to effectively remove the outer layers of materials, such as the skins of fruits and vegetables and certain industrial coatings [34]. This technique is innovative due to its unique advantages, such as operating without heat or mechanical force, which prevents blunt damage to the materials. Secondly, the water jet peeling device exhibits outstanding penetration abilities, allowing for accurate and effective removal of produce skins while preserving the underlying material. Furthermore, the technology exhibits remarkable adaptability, allowing it to process materials with higher hardness levels and complex geometries, making it a versatile and environmentally friendly solution for diverse surface treatment applications [35].

**FIGURE 5:**
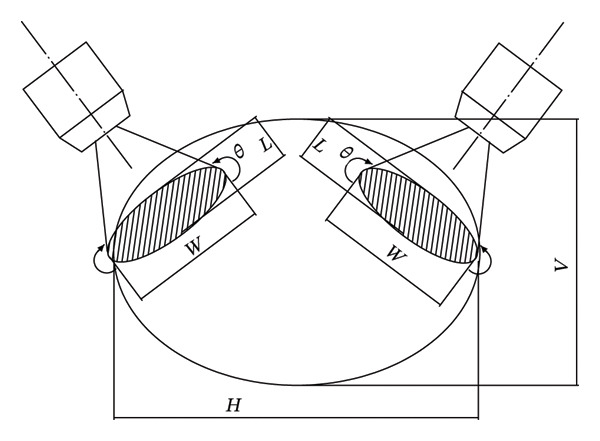
Water jet action on the surface of the lotus seed. Reproduced with permission from Ref. [36].

Xieqing et al. [36] designed a fresh lotus seed peeling machine based on the principle of water jet peeling. The machine uses a clamping device to constrain the lotus seed and drive the lotus seed to rotate around its own axis and then uses two symmetrical water jets to hit the surface of the rotating lotus seed to complete the peeling process. The peeling principle is to rely on the friction with the roller and the water pressure removal when the water jet hits the countercurrent on the surface of the lotus seed (see Figure 5). In order to achieve complete peeling, the parameters are optimized, the water jet pressure is 0.7 MPa, the water jet inclination angle is 18°, the peeling rate of the pulley diameter 68 mm is 92.63%, the damage rate is 1.24%, the cap head rate is 2.72%, and the edge opening rate is 0.21%.

Key experimental parameters influencing water jet peeling performance include water jet pressure, flow velocity, nozzle‐to‐surface distance, injection angle, material properties, and surface geometry [34]. Peeling effectiveness is largely determined by the pressure of the water jet. Higher pressures improve skin removal but can also increase the potential for substrate damage. The speed of flow greatly influences the intensity of impact; too little speed results in incomplete peeling, while too much can deform the material. To ensure efficient peeling, optimal nozzle positioning is defined by a proper distance (typically 5–15 mm) and an injection angle (usually 30°–60°) for effective energy concentration. Uniform peeling is harder to achieve on complex surfaces with irregular geometries, often requiring specially configured nozzles [36].

### 2.5. Rocker Vibration Peeling

**FIGURE 6:**
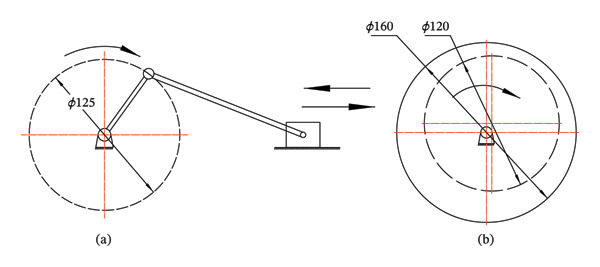
Two types of rocking vibration structures: (a) crank‐slider mechanism and (b) eccentric rotating mechanism. Reproduced with permission from Ref. [37].

The rocker vibration peeling mechanical structure can efficiently and quickly remove the skin of fruits and vegetables, while maintaining the freshness of the pulp (see Figure 6). By adjusting the parameters such as vibration frequency, amplitude, and rocker angle, the characteristics of different fruits and vegetables can be optimized to ensure the best peeling effect and the improvement of production efficiency [37, 38].

### 2.6. Structure of Pomegranate Peeling Machinery

**FIGURE 7:**
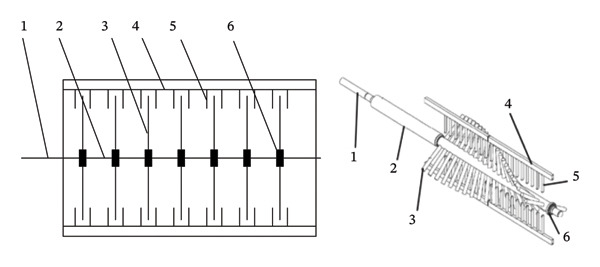
The schematic diagram of the comb brush structure. Reproduced with permission from Ref. [41]. 1: Spindle, 2: screen shaft, 3: impact round rods, 4: stainless steel reinforced square tube, 5: flat comb, and 6: bearing.

At present, the mechanical peeling method of pomegranate peel and seed extraction is mainly by crushing and squeezing and knocking [39]. The method of crushing and extrusion is to crush the whole pomegranate directly into blocks, and hit the pomegranate seeds under the action of a baton [40]. The crushing mechanism is widely used in fruit and vegetable processing machinery. Wetzstein et al. [41] designed a pomegranate peeling and seed picking machine based on the principle of human hand skin friction. Under the continuous strike of the strip tooth comb and sheet tooth comb, the fruits and vegetables are gradually dispersed. Finally, the peel and diaphragm are discharged from the lower end of the sieve and fall into the peel diaphragm discharge port. The crushing mechanism is mostly gear meshing transmission, as shown in Figure 7. The seed extraction mechanism is connected to the motor by a rotating shaft, and the rotating shaft is equipped with a striking rod [42]. The length of the shaft, the speed of the gear, and the number of striking rods affect the length and number of pomegranates [43]. The size of the striking rod, the number of striking rods, and the speed of the rotating shaft all significantly affect the seed extraction results of pomegranates. The length of the shaft, the speed of the gear, and the number of installed striking rods play an important role in the efficiency of pomegranate seed extraction. The core of the seed picking machine with crushing and striking method is to simulate the process of rubbing pomegranate seeds by hand [44].

The seed extraction method of crushing and extrusion is used because the complete pomegranate is directly broken into blocks, and the pomegranate is broken after being subjected to large pressure, which leads to a high damage rate of pomegranate seeds, and there are a large number of impurities and bacteria in the calyx [45]. At the same time, the pomegranate seeds have been polluted, and the subsequent separation device cannot adjust the gap, and there will be incomplete separation of peels and seeds [46].

**FIGURE 8:**
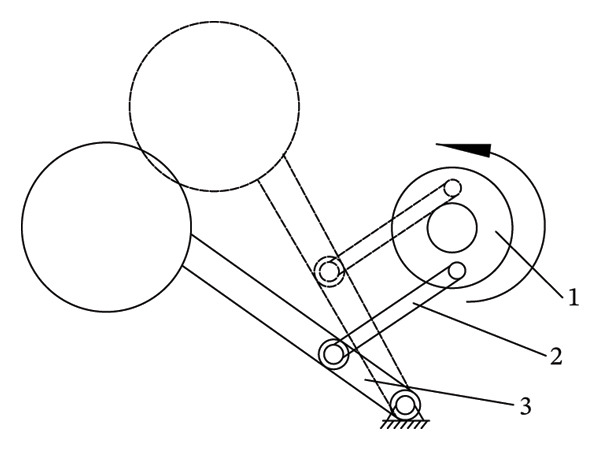
Reciprocating striking mechanism. Reproduced with permission from Ref. [47].

The main structure of the round‐trip knocking pomegranate seed picking machine is to hit the half‐cut pomegranate. The knocking pomegranate seed picking machine is mostly a crank rocker rotary mechanism (see Figure 8). When the knocking frequency is 15–25 Hz and the amplitude is 5–8 mm, the damage rate is low. When the knocking frequency is > 25 Hz, the fracture efficiency at the junction of the fruit membrane and the pulp is improved, but the high frequency leads to the accumulation of elastic deformation energy and the increase in the damage rate [48, 49].

**FIGURE 9:**
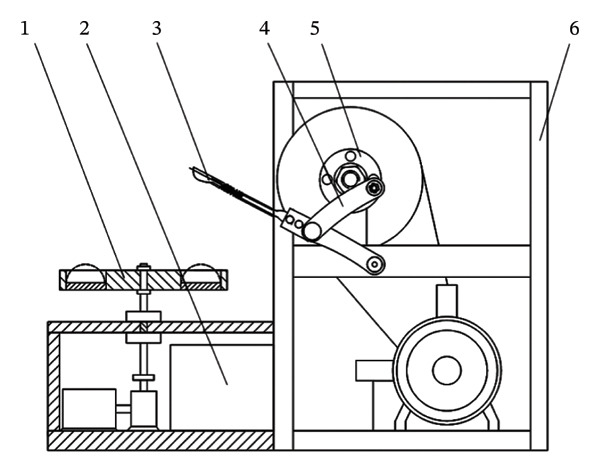
Schematic diagram of the working structure of a vibrating pomegranate dehulling device. Reproduced with permission from Ref. [47]. 1: Pomegranate vibrating bowl, 2: collection bin, 3: striking device, 4: crank‐rack mechanism, 5: transmission system, and 6: frame.

Sanchez Angel et al. [47] improved the above problems and designed a pomegranate seed picking machine that can perform multipoint hitting on the cut half pomegranate. By hitting different positions of the cut half pomegranate, and optimizing the hitting frequency, the amplitude of the hitting rod group is 50 mm when the single torsion of the hitting shaft is 60° so that the connection between the pomegranate seed and the tire seat is broken, and the pomegranate seed is dropped (see Figure 9). The main influencing factors of this method include the position of the hit and the rotation speed and frequency of the knocking rod. The rotation speed and frequency of the knocking rod have a great influence on the success of the seed. The shape structure of the half‐cut pomegranate was analyzed. It was found that the knocking effect was the best at the half height of the pomegranate [50].

Although the round‐trip knocking method maintains the integrity of pomegranate peel and reduces the crushing rate of pomegranate seeds, it is bound to hinder the falling of pomegranate seeds because of the support of the rack for cutting semipomegranate, and some pomegranate seeds close to pomegranate peel are tightly wrapped by the interfloral membrane (mostly white film) in pomegranate, which is difficult to knock off, and the separation rate of pomegranate peel and seeds is not ideal. Subsequent improvements can adjust the distribution of the striking shop from the parameter matrix of the striking array or change the contact range between the striking stick and the pomegranate, the hexagonal or triangular striking stick striking the pomegranate, and the multifrequency working together to improve the pomegranate processing machinery [51].

The effects of three parameters—crank length, crank rotational speed, and vibrating feeder speed on the target response values of pomegranate seed removal rate and damage rate (see Table 1). The significance level *p* for the target response pomegranate seed removal rate is < 0.0001 and that for the damage rate is < 0.0001 (*p* < 0.01), indicating that these three parameters exert an extremely significant influence on the target response values.

**TABLE 1:** Pomegranate dehulling test results.

No.	Crank length (mm)	Motor speed (r·min^−1^)	Vibration disc speed (r·min^−1^)	Response value	
				Seed yield rate (%)	Damage rate (%)
1	50	100	20	88.69	6.12
2	50	140	40	90.60	3.71
3	50	140	20	93.85	3.53
4	50	100	40	80.90	5.90
5	50	120	30	93.77	3.61
6	50	120	30	93.03	3.35
7	50	120	30	94.09	3.75
8	50	120	30	93.29	3.56
9	50	120	30	92.92	3.61
10	40	100	30	88.20	6.08
11	40	120	20	91.93	6.16
12	40	140	30	99.15	3.55
13	40	120	40	82.65	4.62
14	60	140	30	96.46	2.31
15	60	100	30	90.66	3.99
16	60	120	20	91.80	3.74
17	60	140	30	87.86	4.11

In order to realize the high efficiency and low loss of mechanized seed extraction of pomegranate, five typical schemes are put forward as shown in Table 2. There are significant differences in key indicators such as operation efficiency and equipment cost. Although the high‐pressure water jet scheme of Scheme 4 has the highest efficiency potential, its “very high” equipment cost, complex water circulation treatment system, and specific requirements for working environment are seriously inconsistent with the budget and conventional conditions of general laboratories. Scheme 2 has the lowest cost of the friction mechanical scheme, but its operation efficiency is obviously low, and it is mainly aimed at peeling. The applicability of complete seed picking may be insufficient, and the technical advancement is limited.

**TABLE 2:** Efficiency and cost comparison of different peeling methods.

Scheme	Efficiency (kg/h)	Equipment cost	Source
Blade cutting	300	High	[21]
Friction peeling	200	Low	[28]
extrusion deformation	300–800	Middle–high	[33]
Water jet	600–1600	Very high	[33]
Vibration seed harvesting	100	Middle–high	[47]

## 3. Pomegranate Internal Structure and Mechanical Properties Testing

### 3.1. Pomegranate Morphological Features

**FIGURE 10:**
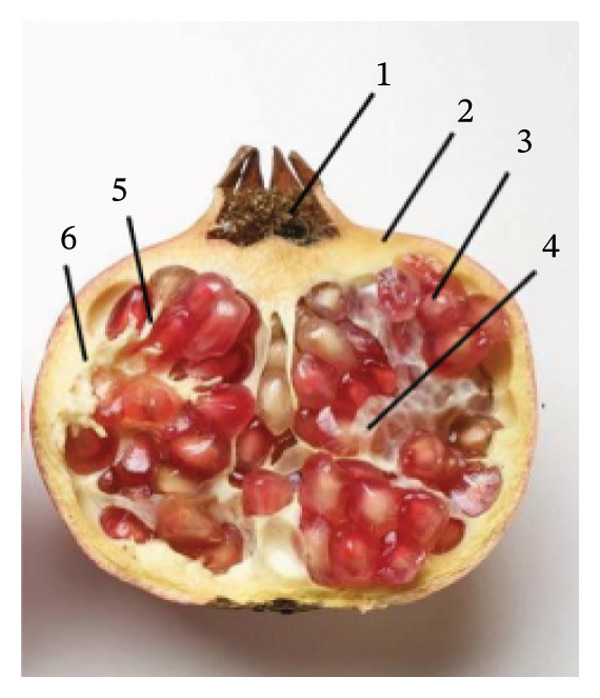
Pomegranate internal structure diagram. 1: Calyx (18–25 mm), 2: peel (1.5–3.7 mm), 3: pomegranate seeds (8.46–10.4 mm), 4: pomegranate membrane (0.1 mm), 5: pomegranate connective pedicel (0.8–1.4 mm), and 6: pomegranate placenta (1.5–3.7 mm).

Fruit morphology was characterized by the shape index, which is used to reflect the shape characteristics of fruit (see Figure 10). The larger the ratio, the longer the fruit is; the value close to 1 indicates that the fruit tends to be spherical [52]. The fruit shape index of pomegranate at maturity is usually close to 1, which is round‐like.

**FIGURE 11:**
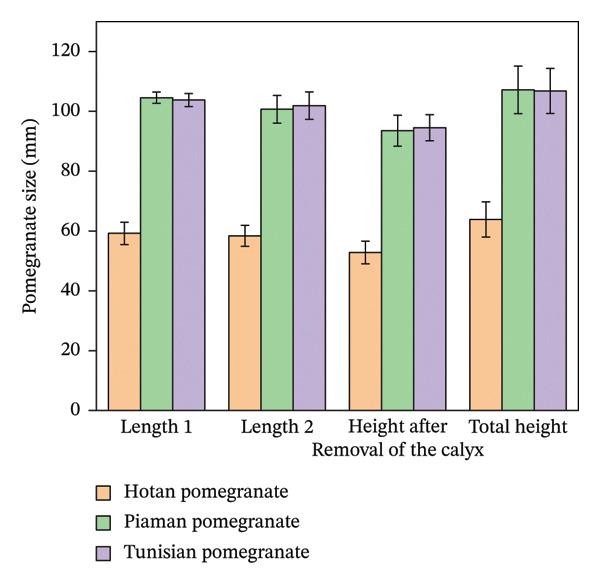
Variations in pomegranate size across Xinjiang.

**FIGURE 12:**
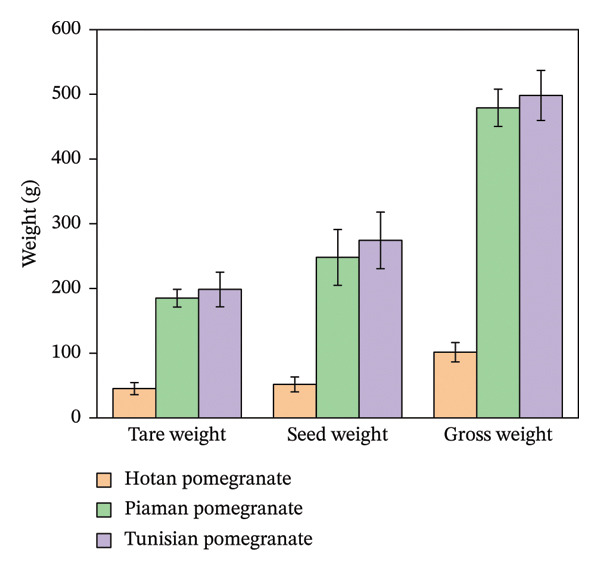
Changes in the weight of various parts of Xinjiang pomegranates.

Pomegranate varieties in the Hotan area of Xinjiang were selected as experimental objects to study the internal structure of pomegranate and the mechanical properties of pomegranate tissue. Twenty pomegranates of each variety were randomly selected for external dimension measurement and pomegranate weighing. The single fruit weight was weighed by an electronic scale (accuracy of 0.01 g). The maximum length (*L*) and maximum diameter (*D*) in the longitudinal direction of the fruit were measured by a digital vernier caliper (accuracy: 0.01 mm). Repeat the measurement three times, and record the mean value. The measurement results are shown in Figures 11 and 12.

According to Mohsenin [55], the fruit geometric mean diameter (Dg, mm) and sphericity (φ) were determined using Equation (1), and the surface area (SF, cm^2^) was determined using Equation (2).

(1) Dg=L×D213/,

(2) φ=DgL.

The main physical parameters of three pomegranate varieties were measured, and the results are shown in Table 3. Tunisian pomegranate is characterized by the heaviest single fruit and has a slightly higher seed yield. Piyaman pomegranate has larger grains and thicker peel. Pomegranate with wrinkled skin is significantly lower than the former two in fruit size and skin thickness, which belongs to small fruits, but the fruit shape is closer to spherical.

**TABLE 3:** Physical characteristics of different pomegranate varieties in Xinjiang.

Cultivars grown	Total weight (g)	Peel weight (g)	Seed weight (g)	Sphericity	Total high (mm)	Seed separation rate (%)	Length of pomegranate seeds (mm)	The thickness of the pomegranate peel (mm)
Piyaman	479.09 ± 28.76	184.99 ± 13.64	248 ± 43.16	0.92 ± 0.02	107.17 ± 7.96	53 ± 18	8.51 ± 0.36	3.71 ± 0.36
Tunisia	498.26 ± 38.65	198.47 ± 26.75	274.33 ± 43.72	0.91 ± 0.02	106.8 ± 7.54	57 ± 19	7.62 ± 0.67	3.21 ± 0.27
He tian	101.47 ± 14.96	45.3 ± 9.41	51.78 ± 11.5	0.94 ± 0.04	52.83 ± 3.78	54 ± 18	8.01 ± 0.56	2.31 ± 0.26

*Note:* Sphericity (*φ*) is a dimensionless parameter.

### 3.2. Measurement of Pomegranate Peel Moisture Content

The moisture content of pomegranate peel is an important factor affecting the mechanical properties of the cutting process (see Table 4). Test samples for pomegranate peel moisture content testing were obtained through random sampling from fresh fruits, with a sample size of 20 per variety. The mature pomegranate fruit is washed in tap water, and then, the pomegranate is peeled manually to retain the pomegranate peel. In the experiment, the peels of the two pomegranate varieties were dried by a dryer. The Grubbs test (*α* = 0.05) was used to exclude outliers, and the moisture content was determined by the wet basis moisture content. Pomegranate peel was longitudinally cut into 6‐mm‐wide test samples and placed on a stainless steel thin layer for drying. The samples were dried in triplicate, averaged, and entered into a multistage drying section in an oven with forced air circulation (drying at 50°C, 60°C, and 70°C and 1.0 ms^−1^ air velocity) [54].

(3) W=G1−G0G1×100%,

where *W* is the moisture content on wet basis (%); *G*_1_ is the mass of wet pomegranate peel (kg); and *G*_0_ is the mass of dry pomegranate peel (kg).

**TABLE 4:** Pomegranate peel moisture content.

Place of origin	Cultivars grown	Sample (%)	Population of samples (%)
Xinjiang Uygur Autonomous Region	Piyaman	62.7 ± 0.54	58.2 ± 0.83
Xinjiang Uygur Autonomous Region	Tunisia	44.1 ± 0.64	48.1 ± 0.75

### 3.3. Mechanical Properties Testing of Pomegranate

The mechanical properties’ test of pomegranate can provide effective physical properties data such as tensile strength and shear strength of pomegranate peel, rupture strength of pomegranate seed membrane, and connection strength between pomegranate seed and pomegranate placenta for pomegranate peel and seed removal machinery so that pomegranate peel and seed removal machinery can be efficiently peeled and seeded in the design while avoiding damage to pomegranate seeds as much as possible, ensuring the efficiency and health safety of the peel process.

**FIGURE 13:**
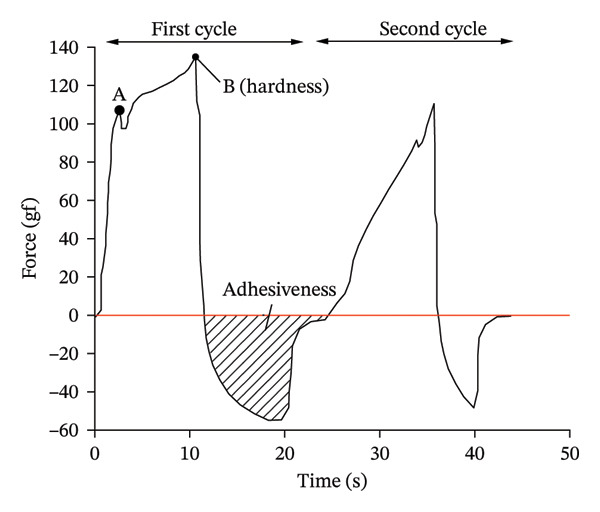
TPA full texture map. Reproduced with permission from Ref. [54].

Figure 13 shows the full texture map under compression. Hardness corresponds to the peak value of the texture map, which is expressed as the yield strength. Adhesiveness corresponds to the shadow area of the image, which is expressed as the elasticity of pomegranate seeds.

Point a in the figure is the biological yield limit, which is the critical point for the initial damage of pomegranate seeds. Near this point, the strain increases and the stress does not increase or decrease slightly, indicating that the local area of the pomegranate seed microstructure begins to break. This limit is a key indicator to determine whether the product is damaged: If the load is always below this limit, the cell system remains intact and the product is not susceptible to corruption.

Point *b* on the curve in the figure is called the biological damage limit, which marks the overall and irreversible damage of the material. After crossing this point, the stress decreases rapidly with the increase of strain, indicating that the macroscopic structure in the load area has been completely deformed. For fruits and vegetables, this failure limit will only be reached after significant plastic deformation. Fruits and vegetables are all viscoelastic materials. Under repeated loads, their tissues will soften and reduce the bearing capacity.

**FIGURE 14:**
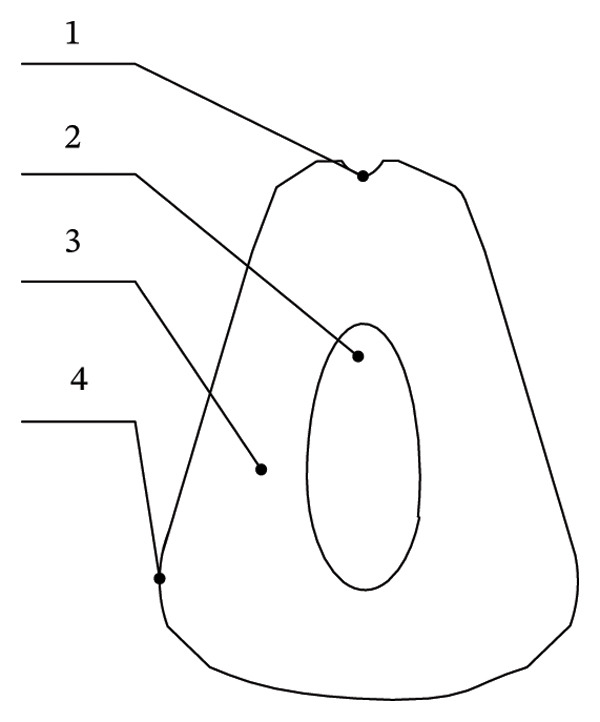
Internal structure diagram of pomegranate. 1: Pomegranate connection part, 2: seeds, 3: aril, and 4: aril fruit membrane.

Figure 14 shows a schematic diagram of the internal anatomy of a pomegranate, clearly illustrating the spatial distribution of the pedicel, seeds, edible arils, and partitioning membranes, thereby providing a morphological basis for the subsequent design of a pomegranate peeling and seed extraction mechanism.

**FIGURE 15:**
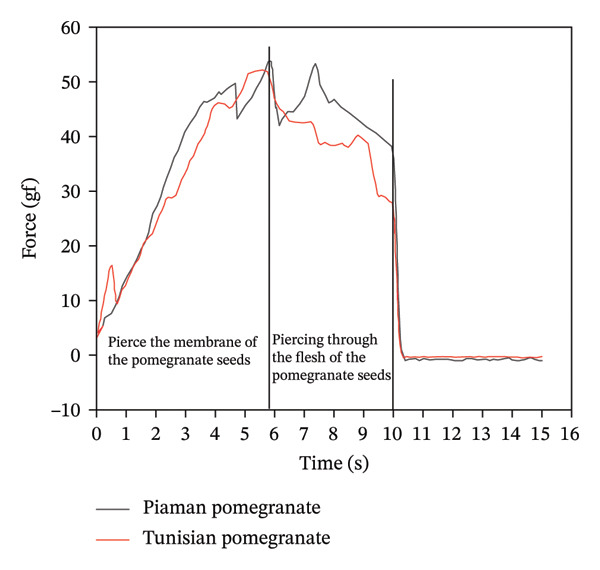
Hardness and strength of TPA pomegranate fruit membranes.

**FIGURE 16:**
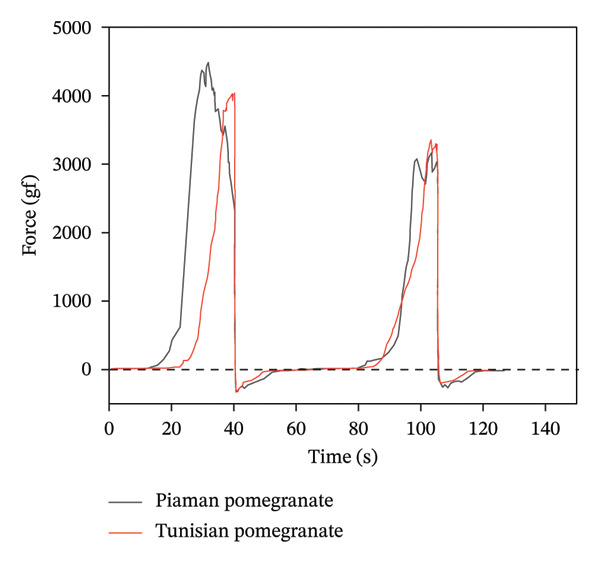
TPA pomegranate peel hardness.

*P*/5 cylindrical probe was used in the experiment. Pomegranate was divided into two groups: Piaman and Tunisian pomegranate. There were 30 samples in each group. Ten grains were randomly selected from each sample for testing. Firstly, height and weight calibration were carried out. Three pomegranate grains with uniform size were closely placed on the stage and measured by the bilateral compression mode. The continuous cycle time interval was 5 s. The speed before and after the test was 4 mm/s, and the test speed was 1 mm/s. The sample was subjected to 50% strain, and the trigger force was 50 g. The total texture characteristics of different pomegranate grains were measured three times in parallel. The maximum peak on the curve can reflect the strength of the pomegranate seed fruit film. Strength of TPA pomegranate fruit membranes is shown in Figure 15. The hardness of TPA pomegranate peel is shown in Figure 16.

Analysis of mechanical property test results for pomegranates and pomegranate seeds reveals that the peel strength significantly exceeds the seed membrane strength (see Table 5). This is attributed to the high cellulose and tannin content within the peel’s cell walls, which are densely arranged, conferring high toughness and strength to the peel. The peel strength of Tunisian pomegranates is slightly higher than that of Piaman pomegranates, at approximately 4276.08 gf.

**TABLE 5:** Textural characteristics of pomegranate seeds from different varieties in the Xinjiang region.

Cultivars grown	Aril hardness (gf)	Peel puncture resistance (gf)	Flesh hardness (gf)
Piyaman	61.86 ± 4.67	4087.25 ± 150.17	53.34 ± 6.02
Tunisia	57.32 ± 3.24	4276.08 ± 101.98	56.36 ± 4.32

## 4. Pomegranate Seed Minor Damage Assessment System

### 4.1. Hyperspectral Detection

In recent years, nondestructive testing techniques for fruit damage have attracted widespread research interest, with numerous scholars conducting in‐depth investigations into this field. Ferrari et al. [57] employ hyperspectral image processing to convert three‐dimensional spectral images into two‐dimensional matrix datasets, subsequently analyzing the images using least‐squares methods to detect damaged areas. In the classification prediction of hyperspectral data, the application of variable selection algorithms significantly improved the classification accuracy on external test sets: achieving 92.42% on the “Golden Delicious” dataset and 94.04% on the “Pink Lady” dataset (see Table 6). In 2014, Rivera et al. [58] used mangoes as samples to compare five methods for segmenting multispectral images at selected wavelengths. Ultimately, the k‐nearest neighbor algorithm was selected to classify intact and damaged pixels, achieving an accuracy rate of 98%. Emmanuel Ekene Okere et al. [59] demonstrated early bruise detection in pomegranate fruits using VNIR and SWIR cameras with visible‐near‐infrared (400–1000 nm) and shortwave infrared (1000–2500 nm) line‐scan mode devices, achieving accuracies of 88.3% and 86.7% for full‐wavelength and selected‐wavelength detection, respectively. Based on the above research findings, develop a pomegranate damage assessment index system.

**TABLE 6:** Hyperspectral comparison of apple bruising over time (700–800 nm). Reproduced from Ref. [55].

Apple varieties	Apple bruising severity	Reflectance	Visual images	Infrared image
Golden delicious	Before bruise	0.84		
	After 5 h	0.76		
	After 3 days	0.64		
	After 6 days	0.63		

Pink lady	Before bruise	0.86		
	After 5 h	0.72		
	After 3 days	0.62		
	After 6 days	0.61		

When pomegranates develop bruises, they are difficult to detect in the early stages, and the bruising becomes more pronounced over time (see Table 7). Hyperspectral imaging can be used to detect damage to pomegranates, with hyperspectral curve images exhibiting different trends reflecting varying degrees of injury [58]. Damage detection in pomegranate seeds can also be performed using hyperspectral imaging. When surface cells of pomegranate seeds are damaged, the reflectance ratio at specific wavelengths decreases significantly [59]. However, under current experimental conditions lacking different fluorescence wavelengths, this method can only serve as a reference tool.

**TABLE 7:** Hyperspectral comparison of pomegranate bruising over time (700–800 nm). Reproduced from Ref. [58].

Pomegranate bruising degree	Reflectance	Visual images	Infrared image
Before bruise	0.58		
After 5 h	0.42		
After 1 day	0.12		

### 4.2. Texture Analyzer Testing

Textural analysis of pomegranate seeds can measure their hardness and elasticity [60]. There are significant differences in the physicochemical properties between intact pomegranate seeds and damaged pomegranate seeds.

**FIGURE 17:**
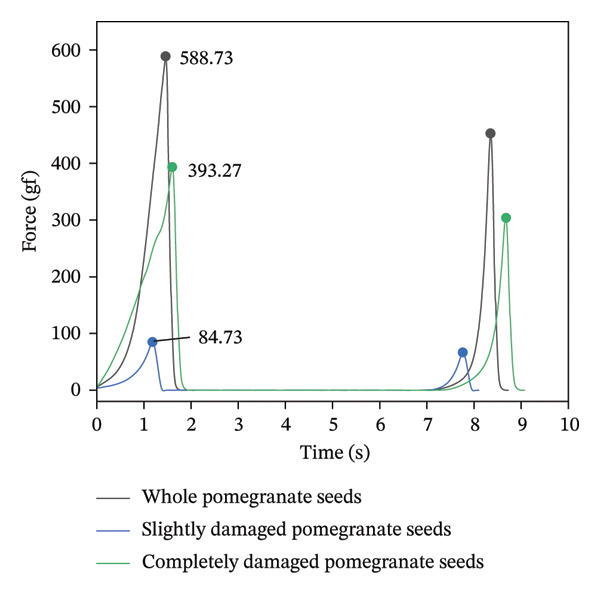
Texture mapping of pomegranate seeds with varying degrees of damage (*P*/100).

As shown in Figure 17, the pulp strength of intact pomegranate seeds is approximately 588.73 gf, while that of seeds with minor surface damage is about 393.27 gf. For seeds with complete damage, the pulp strength is approximately 84.73 gf.

The reasons for the differing trends observed in pomegranate seeds with varying degrees of damage during texturometer testing are as follows: After damage, the seed coat of pomegranate seeds ruptures [61]. Pressure causes the cell walls to rupture, allowing cellular sap to leak out, thereby reducing the overall strength and elasticity of the pomegranate pulp. After complete damage, pomegranate seeds undergo significant morphological changes: Numerous seeds rupture, releasing juice, causing the seeds to shrivel [62]. The hardness of the seed pulp and the elasticity of the seed membrane are significantly reduced. Minorly damaged pomegranate seeds retain most of their intact cells, resulting in little reduction in pulp firmness or membrane elasticity [63].

### 4.3. Pomegranate Fruit Quality Assessment

Five experimental groups were selected, with 10 pomegranates randomly chosen from each group. The seeds from each pomegranate were collected, washed, and bagged. Their weight was measured, and the average value for each group was recorded, denoted as *Q*_11_, *Q*_12_, *Q*_13_, *Q*_14_, and *Q*_15_. During the experiment, select five test groups. For each group, collect the pomegranates from the collection bins, place them in bags, wash them thoroughly, and weigh them, denoted as *Q*_21_, *Q*_22_, *Q*_23_, *Q*_24_, and *Q*_25_, respectively. Place damaged pomegranates from the collection bin into bags, wash them thoroughly, and weigh them, recorded as *Q*_31_, *Q*_32_, *Q*_33_, *Q*_34_, and *Q*_35_, respectively. The calculation formula is

(4) Q¯ij=15∑i=13∑j=15Qij,μ=Q¯2jQ1j×100%,ν=Q¯3jQ2j×100%.

Here, *μ* is the pomegranate dehulling efficiency (%); *ν* is the pomegranate damage rate (%); *Q*_1*j*_ is the average mass (g) of all seeds within each pomegranate before the experiment; *Q*_2*j*_ is the average mass (g) of pomegranate seeds collected in the collection box within each group after the experiment; and *Q*_3*j*_ is the mass of damaged pomegranate seeds collected in the collection box within each group after the test (g). According to the < pomegranate quality grading > standard, the detection threshold for minor damage to pomegranate seeds is set at *v* < 5% [64]. If the total damage rate of pomegranate seeds after extraction is less than 5%, the seeds can be considered to have sustained only minor damage during mechanical processing.

## 5. Pomegranate Peeling and Seed Extraction Scheme Exploration

### 5.1. Scheme Exploration

Pomegranate peeling machines currently available in the market have difficulty adapting to fruits of various sizes. Despite the development of mechanized peeling methods such as cutting tools, air pressure, friction, and vibration, they face major adaptability issues, especially regarding flesh damage, incomplete peeling, and inefficient seed removal. This restriction frequently leads to less effective peeling and can harm the fruit’s internal structure, especially the flesh [65].

The design of pomegranate peeling and seed removal machines should prioritize precise adjustments based on the fruit’s mechanical properties and physical variations. Integrating modern intelligent control technologies with flexible cutting techniques is essential to develop peeling solutions that offer strong adaptability, high efficiency, and maximal flesh protection. This approach should be central to the design of next‐generation pomegranate processing machines [66].

### 5.2. Pomegranate Seed Extraction Plan Design

#### 5.2.1. Overall Design of Pomegranate Processing Equipment

**FIGURE 18:**
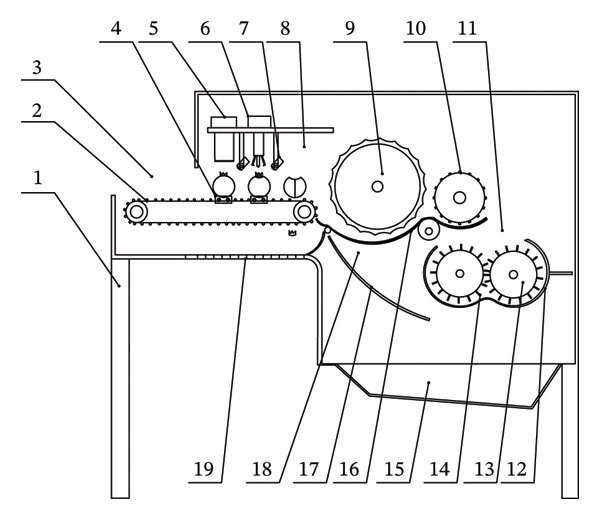
Overall schematic diagram of the organization. 1: Housing, 2: conveyor belt, 3: feeding port, 4: pomegranate fixation seat, 5: calyx removal unit, 6: segment separation unit, 7: auxiliary positioning camera, 8: crushing preprocessing unit, 9: roller, 10: seed extraction wheel, 11: connection channel, 12: circular hole mesh, 13: rotating shaft, 14: impact block, 15: pomegranate seed collection port, 16: adjustable mesh screen unit, 17: pomegranate seed collection auxiliary channel, 18: seed extraction unit, and 19: calyx collection channel.

In view of the above problems in the process of pomegranate seed extraction, a new type of peeled seed structure design and preprocessing treatment research was carried out. A seed extraction device with adjustable kneading gap that can remove the calyx pretreatment of pomegranate was designed and developed, and the peeled pomegranate granules were hygienic and safe (see Figure 18).

**FIGURE 19:**
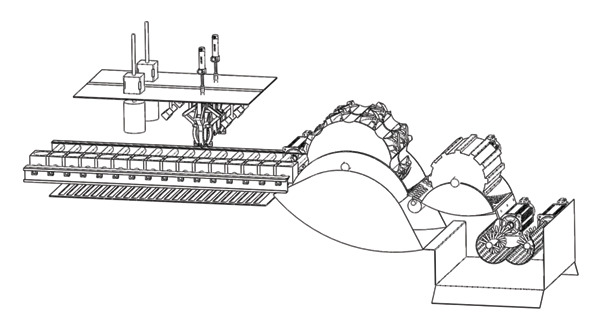
3D rendering of the overall equipment design.

Pomegranate enters the equipment through the feed port and is first transported by the conveyor belt to the crushing pretreatment unit. The calyx and fruit petals of pomegranate were treated by the calyx removal unit and petal division unit, respectively, and the treated pomegranate entered the seed extraction unit. The rollers come into contact with the pomegranates via a layer of elastic rubber and concave grooves; the seeds are separated through the action of rotation and agitation, whilst the seed‐separating wheel further enhances the efficiency of the separation process. The adjustable‐spacing screen unit optimizes the separation effect by adjusting the screen spacing, and the striking blocks of the shaft unit work together to peel the peel. Finally, pomegranate seeds enter the pomegranate seed collection port through the sieve and collection channel, and the peel is discharged through the discharge port (see Figure 19).

#### 5.2.2. Pomegranate Pretreatment System

**FIGURE 20:**
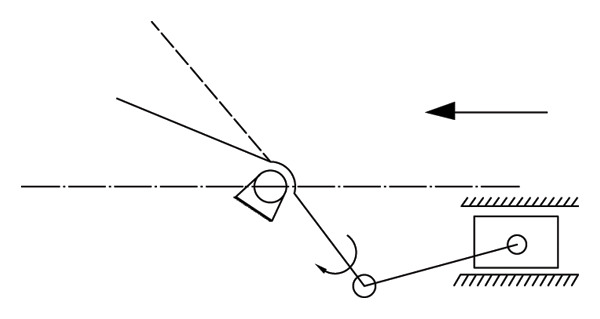
Schematic diagram of the pretreatment structure.

**FIGURE 21:**
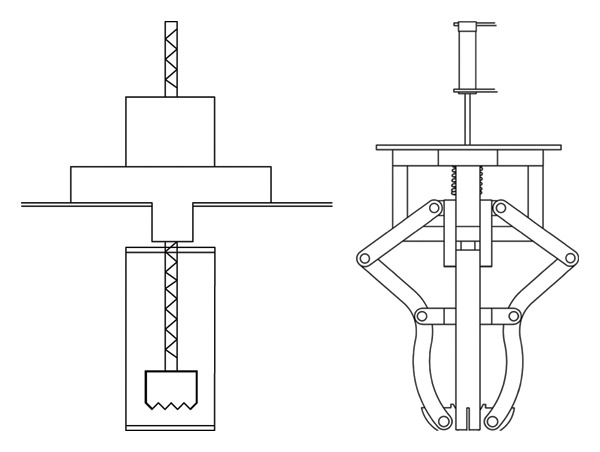
Pomegranate seed system schematic diagram.

The core equipment for the calyx removal process is the circular calyx removal blade. During operation, guided by the visual positioning of an auxiliary camera, the blade precisely locates at the top of the pomegranate calyx. At this point, the lead screw motor activates, driving the cutting blade to rotate downward in a vertical direction. The ring‐like cutting blade evenly cuts the calyx part at the top of the pomegranate, providing a smooth and efficient trimming process. The blade’s rotation during operation effectively and cleanly removes the calyx, with a 98% success rate in complete calyx removal. The process ensures that the calyx is entirely detached from each pomegranate, setting it up for the next steps of peeling and segmenting. The splitting unit’s crucial element is the cutting blade operated by a linear pneumatic motor. When the cylinder motor starts, the blade moves downward, adhering to the pomegranate’s skin curve and making precise cuts on the fruit’s surface. Based on the given process specifications, the cutting depth of 1.5–4 mm is set by altering the blade’s protrusion length through adjusting the tool’s mounting position [67] (see Figure 20). Ensuring the pomegranate skin is properly scored helps in the crushing process and prevents harm to the seeds inside. This method involves scoring the pomegranate skin, which results in several slits that make it easier to break into smaller parts during the next crushing step. The scoring process decreases energy use and material waste during crushing, thus improving production efficiency (see Figure 21).

**FIGURE 22:**
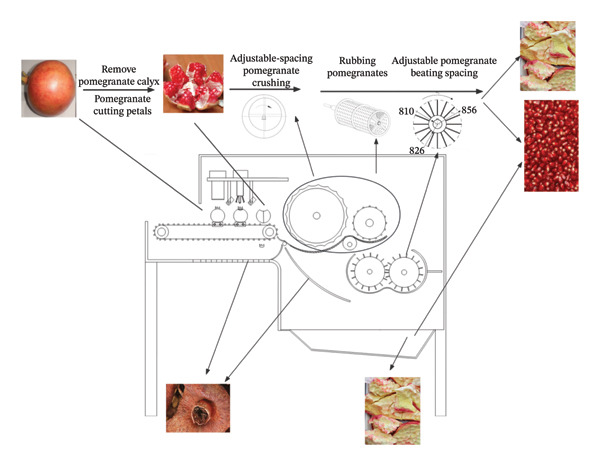
Workflow diagram for pomegranate seed extraction equipment.

The calyx removal process removes the calyx part of pomegranate by precise cutting technology, which lays a foundation for subsequent processing. The peeling process is driven by the cylinder to accurately scratch the crack on the surface of the pomegranate. The whole pretreatment process realized the efficient and accurate processing of pomegranate and provided good preparation for the subsequent crushing, extrusion, and other processes (see Figure 22). These two processes not only improve the processing efficiency of pomegranate but also maximize the integrity of the fruit and reduce the loss in the processing process.

#### 5.2.3. Pomegranate Seed Extraction System

**FIGURE 23:**
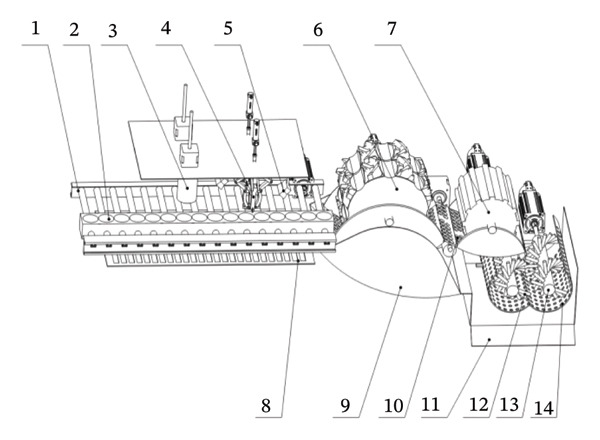
Schematic diagram of the pomegranate seed extraction system. 1: Conveyor belt, 2: pomegranate fixation seat, 3: calyx removal unit, 4: segment separation unit, 5: auxiliary positioning camera, 6: roller, 7: seed extraction wheel, 8: calyx collection channel, 9: connection channel, 10: adjustable mesh screen unit, 11: pomegranate seed collection port, 12: impact block, 13: rotating shaft, and 14: circular hole mesh.

Pomegranate seed extraction system is the core part of the whole pomegranate processing process, which aims to take out the seeds in pomegranate efficiently and accurately through a series of mechanized operations. The system not only requires efficient separation of pulp and seed but also requires maximum preservation of the integrity and quality of pomegranate seeds, while minimizing the loss of pulp (see Figure 23).

This device employs a three‐stage progressive flexible separation architecture, comprising a pretreatment roller unit, an adjustable‐spacing vibrating screen unit, and a dynamic crushing unit. After the calyxes and outer skin are removed, pomegranates are moved to the roller unit using a conveyor belt. The unit features a primary separation chamber with an adjustable screen spacing of 15–25 mm, enabling the gentle separation of pomegranate seeds via progressive compression [41].

During the first stage of separation, after the pomegranate is opened along the precut lines on its skin, around 68%–72% of the seeds go straight through the screen into the collection tray. The inner surfaces of the opened pomegranate segments have seeds that remain attached. Next, the procedure moves to the secondary separation phase, where the seed‐pushing wheel transports remaining pomegranate pieces to the dynamic crushing unit. With asymmetrically meshed discs and an adjustable vibration frequency of 10–25 Hz, this unit produces a controlled vibratory force that increases the yield of intact seeds.

In the concluding screening phase, the mixed material is processed through a double‐layer vibrating screen featuring rectangular openings of 8.5 by 17.5 mm. The integrity of pomegranate seeds has been significantly enhanced, while their processing quality has also been markedly improved, resulting in a substantial increase in the quality of the final product.

## 6. Conclusion

The article delivers an extensive review of the developments in mechanized technologies for peeling fruits and vegetables. Recently, there have been significant strides in research related to mechanized technology for this task [70]. The investigation categorizes widely used peeling equipment at home and abroad, assessing their mechanisms and efficiency. The capability to control equipment damage and the degree of automation are identified as major factors affecting application effectiveness. Research has shifted focus to creating adaptable devices that improve processing with minimal fruit damage, addressing issues found in traditional methods. In recent years, flexible and adjustable fruit processing equipment has become a research focus. To determine the optimal pomegranate peeling method, various techniques were comprehensively evaluated, including knife cutting, friction peeling, crushing and squeezing, water jet peeling, and rocker vibration peeling. With a damage rate of less than 5%, the noncontact waterjet stripping method has become quite popular, even though it involves a relatively high cost for equipment [71]. Researchers have proposed various mechanical designs for seed extraction, including methods such as crushing and squeezing, as well as reciprocating percussion. They found that reciprocating seed extraction devices based on epidermal friction principles and mechanical impact separation systems demonstrated superior performance in preserving seed integrity. Additionally, they conducted in‐depth studies on the fruit structure and physiological characteristics of pomegranates, including statistical analysis of morphological parameters, measurement of the epidermal moisture content, and experiments on the mechanical properties of pomegranate seeds. These investigations have laid a critical technical foundation for advancing the sustainability of the pomegranate deep‐processing sector. However, the field still faces multiple challenges: Most advanced equipment remains in the laboratory stage, with limited technological maturity. The complexity of equipment maintenance has hindered the adoption and promotion of this technology among small‐ and medium‐sized enterprises. The current solution lacks adaptability to changes in raw material properties and has limited versatility. These factors collectively hinder the large‐scale industrial application of the technology.

In summary, within the field of agricultural processing machinery, there is a need to design new peeling solutions to enhance the positioning capability and shape adaptability for peeling fruits and vegetables. Issues such as fruit and vegetable damage, sorting errors, and low efficiency arising from manual processing, alongside challenges like achieving accurate and damage‐free automated processing, require urgent solutions.

## Author Contributions

Enlong Zhu: Conceptualized and evolved the overarching research goals and theoretical framework; involved in drafting the manuscript or revising it critically for important intellectual content.

Jinkun Ran: Annotated, curated, and maintained research data with traceable management protocols; drafted the initial manuscript including substantive translation tasks; presentation of the published work, specifically visualization/data presentation.

Li Li and Junwei Zhu: Preparation, creation, and presentation of the published work by those from the original research group, specifically review, commentary, and revision.

## Funding

This work was financially supported by the Key R & D Project of Xinjiang Uygur Autonomous Region (No.: 2022B02030).

## Conflicts of Interest

The authors declare no conflicts of interest.

## Data Availability

The data that support the findings of this study are available from the corresponding author upon reasonable request.
